# Practical assessment of risk of VILI from ventilating power: a conceptual model

**DOI:** 10.1186/s13054-023-04406-9

**Published:** 2023-04-20

**Authors:** John J. Marini, Lauren T. Thornton, Patricia R. M. Rocco, Luciano Gattinoni, Philip S. Crooke

**Affiliations:** 1 Department of Pulmonary and Critical Care Medicine, University of Minnesota, Minneapolis/St Paul, MN USA; 2 Laboratory of Pulmonary Investigation, Carlos Chagas Filho Biophysics Institute, Federal University of Rio de Janeiro, Rio de Janeiro, Brazil; 3 Department of Anesthesiology, University of Göttingen, Göttingen, Germany; 4 Department of Mathematics, Vanderbilt University, Nashville, TN USA

**Keywords:** Ventilator-induced lung injury, Energy, Power, Mathematical model, Lung protection

## Abstract

At the bedside, assessing the risk of ventilator-induced lung injury (VILI) requires parameters readily measured by the clinician. For this purpose, driving pressure (*DP*) and end-inspiratory static ‘plateau’ pressure ($P_{s}$) of the tidal cycle are unquestionably useful but lack key information relating to associated volume changes and cumulative strain. ‘Mechanical power’, a clinical term which incorporates all dissipated (‘non-elastic’) and conserved (‘elastic’) energy components of inflation, has drawn considerable interest as a comprehensive ‘umbrella’ variable that accounts for the influence of ventilating frequency per minute as well as the energy cost per tidal cycle. Yet, like the raw values of *DP* and $P_{s}$, the absolute levels of energy and power by themselves may not carry sufficiently precise information to guide safe ventilatory practice. In previous work we introduced the concept of ‘damaging energy per cycle’. Here we describe how—if only in concept—the bedside clinician might gauge the theoretical hazard of delivered energy using easily observed static circuit pressures ($P_{s}$ and positive end expiratory pressure) and an estimate of the maximally tolerated (threshold) non-dissipated (‘elastic’) airway pressure that reflects the pressure component applied to the alveolar tissues. Because its core inputs are already in use and familiar in daily practice, the simplified mathematical model we propose here for damaging energy and power may promote deeper comprehension of the key factors in play to improve lung protective ventilation.

**Supplementary Information:**

The online version contains supplementary material available at 10.1186/s13054-023-04406-9.

## Motivation for a developing a mathematical model of damaging energy

Current concepts regarding ‘lung protective’ ventilation of acute respiratory distress syndrome (ARDS) center on not repeatedly exceeding the upper limits of tolerable tissue strain. The clinical term ‘mechanical power’, which incorporates all dissipated (e.g., ‘resistive’) and conserved (‘elastic’) energy components of each inflation cycle over a minute’s span, has drawn considerable interest as a comprehensive ‘umbrella’ variable that accounts for the influence of ventilating frequency per minute as well as the energy cost per tidal cycle [1, 2]. In fact, numerical injury thresholds for the cumulative inflation energy delivered per minute (a valid but atypical form of ‘power’) have been suggested from both experimental and clinical data [3, 4]. By itself, however, the absolute numerical value of such power alone may not carry sufficiently precise information to always guide safe ventilatory practice. In previous work we introduced the concept of ‘damaging energy per cycle’ to highlight innate vulnerability and tissue strain as key determinants of that limitation [2, 5].

The underlying vulnerability to parenchymal damage is clearly important; for example, power values well beyond those encountered clinically are applied by the respiratory muscles to move the healthy lung during vigorous exercise without inflicting overtly detectable injury [6]. Moreover, whatever the innate strength or vulnerability of the individual’s lung tissue, it seems unlikely that a given level of ‘power’ applied with high frequency but constrained tidal volumes, modest transpulmonary pressures, and low parenchymal strains carries the same risk as that same power value does when delivered at lower frequency, but higher tidal volumes, transpulmonary pressures and strains [5].

At the bedside, assessing the risk of ventilator-induced lung injury (VILI) requires ventilating frequency as well as certain parameters of the single inflation cycle that are readily measured by the clinician. Indeed, tidal volumes and indicators derived from static pressures measured at the airway opening during passive inflation have been associated with key clinical outcomes [7].

Clinicians customarily partition the total airway pressure into resistive and conserved (elastic) components by stopping flow at end-inspiration, with the static pressure recorded in the absence of flow believed to correlate best with maximum tissue stresses and VILI risk that occur under dynamic conditions. This somewhat imprecise partitioning and association, though reasonable, remains the only one currently available at every bedside.

Averaged values of airway inflation pressures in the absence of muscle effort, such as driving pressure (*DP*) and end-inspiratory static ‘plateau’ pressure ($P_{s}$), are unquestionably useful and help guide clinical decisions [8, 9]. Yet, the mechanical connection between DP and injury is less well understood and appreciated. While transpulmonary pressure is influenced by the series-coupled chest wall and generates the force applied to lung units, its influence is often discounted or even ignored during spontaneous efforts. Whether ventilation is passive or active, however, the local stresses actually applied to different alveolar units vary from site-to-site within any lung. Because a single airway pressure such as DP measured at the airway opening that is deemed to be safe ‘on average’ might prove hazardous to some vulnerable regions of that same lung, *relative* safety might be a term more applicable to any such measurement made regarding such a mechanically diverse and gravity-influenced structure. If so, only a fraction (large or small) of the presumed ‘safe’ energy and power may actually be well tolerated. In this context, the key role of the peri-alveolar vascular environment in susceptibility to inflation injury and that of ventilating frequency must not be discounted; even an initially small anatomic zone at high risk for injury may eventually extend to damage a wider area when intolerable forces are applied often enough.

**Fig. 1:**
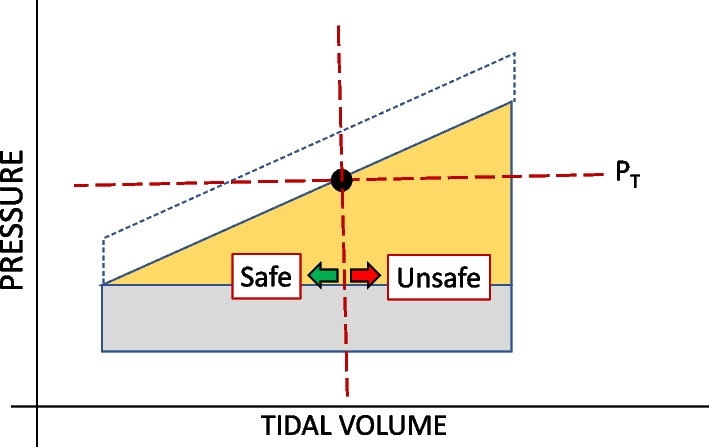
Partitioning of elastic inflation energy (a pressure × volume product and area) by a threshold pressure ($P_{t}$, solid dot) into components that are potentially non-injurious (‘safe’) and hazardous (‘unsafe’)

Because damage cannot occur without its application, energy is central to understanding the VILI mechanism. Following current clinical practice and using clinical nomenclature, we previously elaborated the concept of ‘damaging *energy* per cycle’ by partitioning the inflation stress at a hypothetical value for conserved (termed ‘elastic’) airway pressure [10]. In this paper we describe how—if only in concept–the bedside clinician might gauge under passive conditions the relative hazard of delivered energy per inflation cycle using easily observed static circuit pressures ($P_{s}$ and positive end expiratory pressure (*PEEP*)) and an estimate of a threshold value of elastic airway pressure ($P_{t}$) that might logically serve to partition total intracycle energy into its conserved and dissipated components [6]. Energy that is truly conserved (elastic) cannot itself have been spent in directly damaging tissue, but it does correlate strongly with the stretch and strain that have the potential to do so under dynamic conditions. (Please see Additional file 1: Part A for further explanation.) In the current extension of that earlier simplified model presented here, inflation energy delivered before this $P_{t}$ is reached we designate to be, on average, the ‘safe’ fraction of the total (Fig. 1). Understanding that local (regional) airway pressure thresholds vary from site-to-site, we use the average and region-relevant threshold pressures to compute the ‘safe’ and ‘hazardous’ proportions of total intracycle energy. Our intent is to help caregivers understand the underlying energetic factors in play when Ps, PEEP and DP are adjusted.

## Rationale for damaging energy estimation

**Fig. 2:**
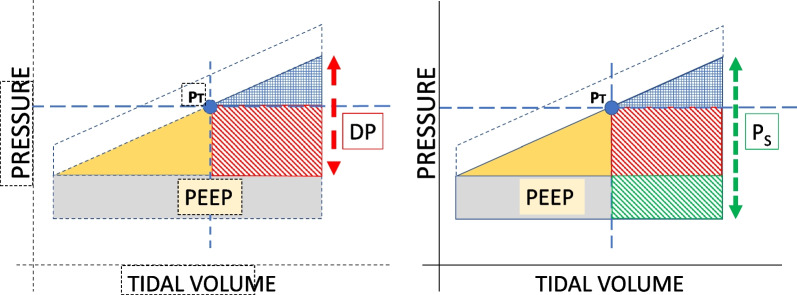
Hazardous energy (crosshatched area) considering all elastic pressure (driving pressure + PEEP) on right panel, or only elastic driving energy on left panel

The clinical term ‘power’ has been defined as the simple product of frequency per minute and total inflation energy per cycle [11]. The latter, therefore, is key to assigning power safety or risk. Following relatable clinical terminology and understanding, in the subsequent discussion we designate tissue level pressures that are not resistive as ‘elastic’. For lung tissue of any vulnerability (fragility), we hypothesize that three clinically measurable factors primarily determine the hazard imposed by the inflation pattern: ventilating frequency, total ‘elastic’ energy delivered per inflation cycle, and proportion of that elastic energy occurring above the hypothetical stress (and strain) threshold that corresponds to the maximally tolerated non-resistive pressure. For any inflation volume, that ‘elastic’ pressure has two measurable components: *PEEP* and the conserved, non-resistive additional pressure needed to deliver the tidal volume against elastance, the inverse of compliance (Fig. 2). At end-inflation, the latter pressure is DP. Because ‘damaging power’ may be considered the simple product of frequency and the hazardous energy per breath, in the subsequent discussion we focus on the energy associated with non-dissipated (‘elastic’) pressures of the individual cycle, which correlates with the potentially damaging strain that occurs under dynamic conditions. For simplicity and clarity, we also ignore the role of chest wall elastance.

## Threshold pressure for damage

The actual threshold pressure, $P_{t}$, that determines the damaging potential for elastic energy *per cycle* is unknown and likely varies–both between patients and within the various regions of the mechanically heterogenous lung [12]. As already noted, two numbers that serve currently as lung protection guidelines for clinical practitioners are the end-inspiratory pressure in the absence of flow, the ‘plateau pressure’, $P_{s}$, and the ‘driving pressure’, $DP = P_{s} - {\text{PEEP}}$ [13, 14]. When the entire breath is considered using observable bedside data during passive ventilation, the numerical upper limits commonly considered ‘safe’ for these parameters in everyday practice are: $P_{s}$ = 30 cmH_2_O and *DP* = 15 cmH_2_O [13, 14]. Fundamentally, however, pressure alone cannot injure—to do that the expenditure of sufficient and potentially damaging energy (a product of pressure and volume) is required. One plausible approach to approximate the latter may be to use the $P_{t}$ as a boundary marker.

Once $P_{t}$ is assigned, the proportion of the total intracycle elastic energy that does *not* exceed that threshold value can be estimated as a ‘safety ratio’ (SR), which we designate to be the quotient of infra-threshold $(P_{t} )$ elastic inflation energy to that of the entire inflation cycle. The complementary *hazardous* energy fraction (HR) bears a simple relationship to SR: ${\text{HR }} = (1 - {\text{SR}})$). Some authors and clinicians consider the only elastic energy component of VILI interest to be the purely dynamic piece (the ‘driving energy’) expended in reaching the potentially hazardous end-tidal *DP* [15]. Alternatively, the elastic energy of concern might also include the PEEP-related static component of pressure, as well as the DP [2, 11]. Here, the $P_{s}$ is the relevant pressure associated with maximum risk, as it helps define the total of ventilation-delivered elastic energy and parallels strain. Therefore, one might set two elastic SRs for each breath: one purely dynamic $\left( {{\text{SR}}_{{{\text{Drive}}}} } \right)$ and one that is all-inclusive $\left( {{\text{SR}}_{{{\text{Elastic}}}} } \right)$. Using these *SR* indicators with a safety threshold for total elastic inflation pressure in mind ($P_{t}$), the delivered breath can be characterized by the proportion of its elastic *energy* that is, on average, ‘safe’ or ‘hazardous’.

If the *SR* for either driving energy or total elastic energy of the *PEEP* and $V_{T}$ combination exceeds the desired *SR* value, new targets for maximally safe *DP*, $P_{s}$ and $V_{T }$ can be calculated. In clinical practice, adjusting *PEEP* may have consequences for hemodynamics and gas exchange. Therefore, again in principle, the linked variables adjusted by the clinician to improve the energetics of VILI risk would likely be tidal volume and *DP* (with frequency fine-tuned as needed to maintain ventilation stable).

## Estimating hazardous tidal energy

Inflation energy, though critically relevant to VILI generation, is seldom measured when ventilating ARDS. Instead, clinicians routinely determine $P_{s}$, *PEEP* and *DP* [14]. These are imprecise and often variable breath to breath during spontaneous efforts. We propose, however, that under passive conditions, the proportion of the elastic energy per breath of the chosen inflation pattern that is potentially hazardous (HR) or safe (SR), can be estimated using these simple inputs alone, together with an arbitrarily assigned ‘threshold’ airway pressure, $P_{t}$. As already mentioned, guided by evidence from randomized trials, *DP* and $P_{s}$ values of 15 and 30 cmH_2_O, respectively, are generally considered as upper bounds for safe ventilation in many ARDS patients. These values, though derived from population-based averages of clinical trials, are incorporated into many bedside protocols for ventilator settings. Yet, disconnected from a solid physiological basis, they are unlikely to prove reliable guides for safe ventilation of every individual—in different patients, damaging energy may be inflicted at numerically higher or lower values. Nonetheless, in the absence of other widely adopted pressure standards, such numbers might be viewed as readily available candidates to use in calculating $P_{t}$ [14–16]. For example, in such an (admittedly imprecise) calculation, $P_{t}$ might be estimated as ≤ 15 cmH_2_O + PEEP.

The pace at which the energy of the single inflation is delivered (intracycle power), as well as the duration of potentially damaging stress per cycle spent above $P_{t}$, is determined by the flow profile [17]. However, as we have previously shown, the same total amount of *elastic* energy or work (W) is required by all flow waveforms to deliver the tidal volume (*V*_*T*_) into a lung with an unchanging compliance ($C$) [18, 19]. That amount of elastic energy is:

$$W_{{{\text{Elastic}}}} = \frac{1}{2}\left( {P_{s} + {\text{PEEP}}} \right)V_{T} = \frac{1}{2C}\left( {V_{T} } \right)^{2} + V_{T} {\text{PEEP}}$$

In theory $P_{t}$, (referenced to zero cmH_2_O) partitions the elastic energy of inflation—either the total elastic energy that includes the PEEP component or the driving elastic energy component that excludes it, which we term *drive energy* $\left( {W_{{{\text{Drive}}}} } \right)$:

$$W_{{{\text{Drive}}}} = \frac{1}{2}P_{s} V_{T} = \frac{1}{2C}\left( {V_{T} } \right)^{2}$$

A geometrical approach indicates that once such a threshold is specified, ‘hazardous’ (> threshold pressure) and ‘safe’ (< threshold pressure) intracycle energy ‘blocks’ can be assigned to the relevant pressure x volume areas that determine intracycle elastic energy (with or w/o PEEP). These relationships are illustrated in Fig. 2 for constant flow but are applicable to any flow waveform. We define the *hazard ratio* (*HR*) as the proportion of the energy–drive $\left( {W_{{{\text{Drive}}}} } \right)$ or elastic $\left( {W_{{{\text{Elastic}}}} } \right)$–that occurs when the elastic pressure in the lung is above a *threshold pressure* ($P_{t} \le P_{s}$). These ratios we designate as ${\text{HR}}_{{{\text{Drive}}}} { }\,\,{\text{or }}\,\,{\text{HR}}_{{{\text{Elastic}}}} {, }$, respectively. In either case, it is the energy fraction delivered in the “hazardous” portion of inspiration. Hence, $0 \le {\text{HR}} \le 1$.

Using the area analogues mentioned above, one can show

$${\text{HR}}_{{{\text{Drive}}}} = \frac{{\left( {2{\text{DP}} + P_{t} - P_{s} } \right)\left( {P_{s} - P_{t} } \right)}}{{{\text{DP}}^{2} }}$$

and

$${\text{HR}}_{{{\text{Elastic}}}} = \frac{{P_{s}^{2} - P_{t}^{2} }}{{P_{s}^{2} - {\text{PEEP}}^{2} }}$$

Here ${\text{DP}} = P_{s} - {\text{PEEP}}$ defines the driving pressure. We note that ${\text{HR}}_{{{\text{Drive}}}}$ depends explicitly on only the driving pressure, plateau pressure, and threshold pressure, while ${\text{HR}}_{{{\text{Elastic}}}}$ depends explicitly on plateau pressure, threshold pressure, and *PEEP*. If the *HR* of the current pattern is greater than thought prudent, adjustments can then be made to *V*_*T*_ and/or *PEEP* (and thereby to $P_{s}$ and *DP*) to achieve the desired proportions of ‘hazardous’ and ‘safe’ energy per cycle.

For given values of ${\text{HR}}_{{{\text{Drive}}}}$, $P_{s}$, and $P_{t}$, we can determine a value of driving pressure $\left( {{\text{Target}}\,\,{\text{DP}}} \right)$ that achieves the desired hazard ratio:

$${\text{Target }}\,\,{\text{DP}} = \frac{{1 - \sqrt {1 - {\text{HR}}_{{{\text{Drive}}}} } }}{{{\text{HR}}_{{{\text{Drive}}}} }}\left( {P_{s} - P_{t} } \right)$$

Alternatively, given values of ${\text{HR}}_{{\text{Elastic }}}$, $P_{t}$, and ${\text{PEEP}},$ we can find a target value for the plateau pressure:

$${\text{Target }}\,P_{s} = \sqrt {\frac{{P_{t}^{2} - {\text{HR}}_{{{\text{Elastic}}}} {\text{PEEP}}^{2} }}{{1 - {\text{HR}}_{{{\text{Elastic}}}} }}}$$

Once these target values for *DP* and $P_{s}$ are known, the necessary adjustment to *V*_*T*_ for acceptably safe tidal energy delivery (${\text{Target}}\,\, V_{T}$) can then be easily computed as

$${\text{Target}}\,\,V_{T} = \left( {\frac{{{\text{Target }}\,\,{\text{DP}}}}{{{\text{DP}}}}} \right)V_{T}$$

$${\text{Target}}\,\,V_{T} = \left( {\frac{{{\text{Target}} P_{s} - {\text{PEEP}}}}{{{\text{DP}}}}} \right)V_{T}$$

The interrelationships among target and input variables are illustrated in Additional file 1: Fig. S2.

If one chooses to work with ‘*safe’* driving and total elastic energy ratios (rather than ‘hazardous’ ones) using the same raw inputs for *PEEP, *$P_{s}$*, and *$P_{t}$*,* a similar process can be used to develop the corresponding expressions. These are derived in the Additional file 1: Part B.

## Estimating regional ${\varvec{P}}_{{\varvec{t}}}$

**Fig. 3:**
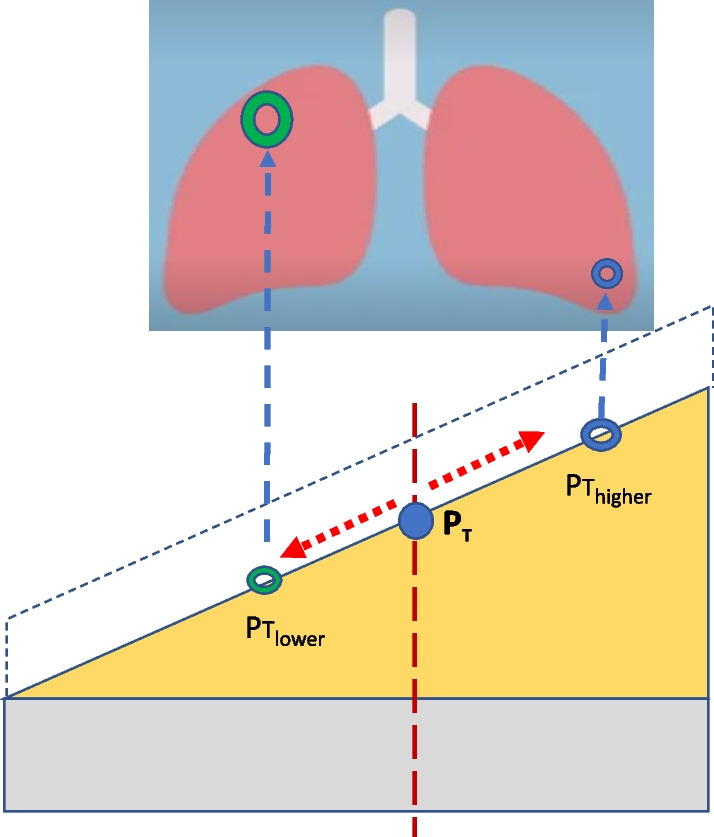
Regional variation of threshold pressure for overstretching as a consequence of transpulmonary pressure. The regional $P_{t}$ of the anatomically non-dependent region (*P*_*t *lower_) is less than the dependent region ($P_{{t\,\,{\text{higher}}}}$)

The stresses that cause local strains within the lung relate to transpulmonary pressure, which for the same airway pressure varies region to region [20]. Damaging regional airway pressures and $P_{t}$ values, therefore, will vary within a given lung, due to gravitational forces, to shape disparities between lung and chest wall, tissue viscoelastance, and to differing local vulnerabilities of tissue caused by inhomogeneous lung injury (due to stress focusing). Considering only the effects of measurable transpulmonary pressure related to tissue stretch (not considering focused stress due to tissue heterogeneity or viscoelastic losses), the threshold airway pressure for damage, i.e., the local $P_{t}$, would vary over a range within the lung. The $P_{t}$ of the more gravitationally non-dependent zones of an evenly injured lung would be lower (indicating greater vulnerability to additional stretch injury than the average value); conversely, the $P_{t}$ of the more gravitationally dependent zones would be higher (indicating less than average vulnerability to overexpansion) (Fig. 3). These different regional thresholds promote or demote the actual transpulmonary stresses that correspond to the measured static airway circuit pressures ($P_{s}$ or DP) applied uniformly to all open units. Note that because the gradient of pleural pressure is less for the prone than for the supine position [21], the width of its $P_{t}$ range of regional vulnerability would be narrower, as well. Regional thresholds and mathematical expressions for use in the corresponding local HRs and Targets are developed in the Additional file 1: Part C.

## Clinical considerations

**Fig. 4:**
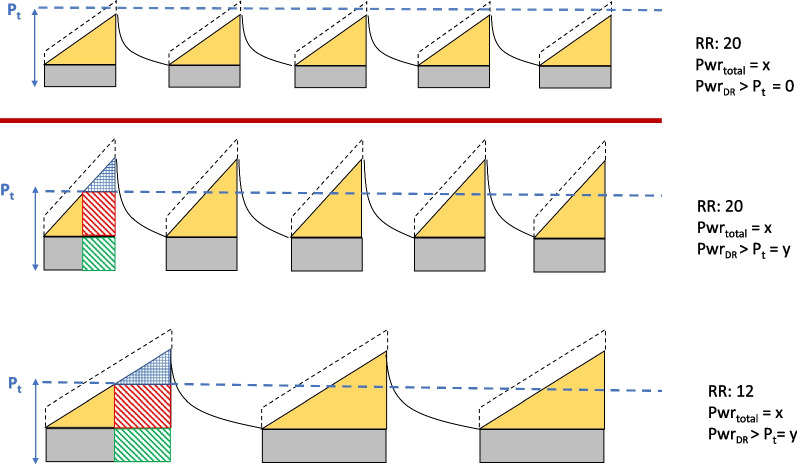
Innocuous Power (top panel) for comparison with two examples of similar total power (×) with nearly equal damaging potential (middle and lower panels). Each individual inflation cycle plots pressure on the vertical axis and time (an analog of inspired volume) on the horizontal axis for the example of constant flow. Damaging power (y) can be delivered by greater frequency of cycles with limited damaging energy (middle panel) or greater damaging energy per cycle delivered at a lower rate (lower panel). Note that whatever the inspiratory flow waveform, whether the same total power is damaging or not depends on both the threshold pressure (*P*_*t*_) and frequency. All damaging elastic energy, $W_{{{\text{Elastic}}}}$, is signified by the sum of all checked plus crosshatched areas. The damaging $W_{{{\text{Drive}}}}$ component of $W_{{{\text{Elastic}}}}$ omits the horizontally crosshatched area that corresponds to PEEP. Driving Power = $W_{{{\text{Drive}}}}$ × frequency; Elastic power = $W_{{{\text{elastic}}}}$ × frequency; *P*_*t*_ = pressure threshold boundary between safe and hazardous inflation energy

Concern for lung protection has gradually evolved from a strategy initially focused on lowering tidal volume to one that currently emphasizes use of safer plateau and driving pressures and avoidance of unnecessarily high ventilating frequency [22]. Yet, considered in isolation, neither high tidal volume delivered into a high-capacity lung nor high elastic pressure applied to units with innately high tissue elastance produces intolerable tidal stretching of parenchymal tissue (excessive strain), the proximate cause of VILI. Conversely, even modest tidal volumes may hyperinflate some vulnerable lung units [23, 24]. Nonetheless, in daily practice set values of $P_{s}$ = 30 cmH_2_O and *DP* = 15 cmH_2_O are commonly considered as the de facto upper threshold pressures to regulate when possible, even if not designated as such [14]. Relatively recent awareness of limitations to numerical guidelines for tidal volume and pressure as well as of the injury potential of repeatedly imposing excessive tidal strains has promoted mechanical energy and power as foci of interest for pattern guidance [1, 2]. When considered in isolation, neither tidal pressures nor power alone are entirely satisfactory as mechanistic explanations for tissue injury [6]. However, because strain is the target and energy is required to strain, their *combination* holds considerable appeal. Specifically, the same cumulative damaging energy per minute (damaging power) may result in VILI, whereas the same total power level delivered with per cycle stress held below the stress threshold might not (Fig. 4). It follows that if the safety or hazard of tidal elastic energy could be estimated from easily monitored data, as modeled in this conceptual exercise, adjustments to its determinants would—at least in principle–better serve the clinician’s goal of avoiding lung injury. The approach offered here is a first and admittedly imprecise attempt to show how that pragmatic goal might eventually be accomplished. While the unrefined conceptual analysis we undertake describes key factors in play and some of their theoretical interactions, we acknowledge that it is only a first endeavor to integrate—and thus better focus—our current lung protective strategies for effective practice.

## Limitations of the model

Although conceptually relatable because it concerns measurable variables (i.e., $P_{s}$, *PEEP*, and frequency) as well as numerical guidelines for dangerous elastic pressures ($P_{t}$) that are both familiar and readily available at the patient’s bedside, the imprecision and practical limitations of such theoretical modeling are clear. Used alone, airway pressures do not allow the forces acting on the lung itself to be teased from those acting on the chest wall. The same concern might be directed toward inflation energetics, as well. Moreover, as discussed in the Additional file 1, measurable pressures correlate with, but do not precisely track either actual tissue stresses or the strains that result from them. Perhaps most importantly, even if conceptually useful, arbitrarily set thresholds for pressure or damaging energy do not identify the ones actually relevant as an injury stimulus. Many cofactors not considered here amplify strain and VILI risk; alveolar geometry, flow pattern and vascular pressures strongly influence micro-mechanics [25–27]. We therefore make no pretense that the simple formulae we present, like their constituent variables that are used currently to guide daily management, are highly precise.

## Conclusion

Despite being inexact and theoretical, the model for damaging energy and power developed here would seem mechanistically plausible and consistent with the most current understanding of VILI causation. Because its core inputs are already in use by many practitioners, such an approach would seem to hold instructive appeal for deeper comprehension and integration of the factors in play to improve lung protective ventilation. We envision that a refinement of such modeling may eventually offer a conceptual framework with actionable clinical utility.

## Supplementary Information

**Additional file 1. Part A:** Relation of Conserved Energy to Damaging Potential. **Part B:** Safety Ratios and Targets.

## Data Availability

Not applicable.
