# Supplementary material for: Practical assessment of risk of VILI from ventilating power: a conceptual model
Source: Crit Care. 2023 Apr 20;27:157. doi: 10.1186/s13054-023-04406-9 (PMC10120146; doi:10.1186/s13054-023-04406-9)
Supplement: Supplementary file 1 — Additional file 1. Part A: Relation of Conserved Energy to Damaging Potential. Part B: Safety Ratios and Targets. [file 13054_2023_4406_MOESM1_ESM.docx]

**Part A: Relation of Conserved Energy to Damaging Potential**

Mechanical inflation energy can be broken down into 3 non-overlapping categories: dissipated, elastic, and deforming (‘and/or damaging’). The elastic component conserved during inflation is stored as potential energy that when released drives exhalation. Because energy can neither be created nor destroyed, an energy component that corresponds to conserved pressure, such as the end-inspiratory static pressure, could not itself have been already spent *directly* in damage. In the existing clinical literature, however, the term ‘elastic’ is applied to the clinically measurable (static) pressures that reflect the component of airway pressure that sustains expansion of lung tissue (e.g., ‘plateau’ and ‘total PEEP’). The component of unrecovered energy that is directly lost in microfractures and tissue deformation in principle, relates more specifically to the clinically unmeasured hysteresis and viscoelastic components that occur during *dynamic* expansion.

Therefore, our pragmatic choice of relating the commonly used clinical term: ‘elastic’ to damaging energy is imprecise but it is highly relevant to VILI. The production of excessive *strain* is the key to parenchymal injury, and while the measurable end-inspiratory static pressure (termed by clinicians the ‘plateau’ pressure for the respiratory system) may not perfectly quantify the forces and energy that generate damage and inflammatory mechano-signaling during its accumulation, it does track them. Likewise, energy that involves driving pressure, the difference between two conserved static (elastic) pressures, reflects incremental tissue stretching energy that may have *signaled* inflammation but cannot itself have been directly spent in damage. It follows that these measured quantities based on static measurements represent conserved pressures that marginally underestimate the total pressures applied to tissue in the process of tissue stretching. The difference between static elastic (conserved) and inflation pressures that act at the tissue level under dynamic conditions corresponds to viscoelastic losses. This small difference is buried within what is usually assigned clinically to the difference between peak and plateau pressures that caregivers use to calculate ‘resistance’. (Figure S1) That hidden pressure difference involves unmeasured energy spent in viscoelastic pressure losses and potentially in the direct infliction of damage by microfractures of extracellular matrix elements.

In summary, energy determined by clinically measurable conserved static pressures does not itself directly quantify injuring energy but is tightly associated with the energy that includes it. In other words, the static energy is conserved *potential* energy and yet tracks the total energy required for the straining that induces damaging microfractures or inflammatory mechano-signaling. Energy must be applied to strain further above baseline, and pressure is a key determinant of that energy. Thus, as described in the main text of this paper, *measurable* pressure may exceed a threshold beyond which additional strain (requiring energy input) is poorly tolerated. These measurable pressures and energies both correlate with tidal stress and stretch. It follows that energy input above the conserved pressure threshold holds the potential for excessive strain. By implication, the so-called ‘elastic’ energy represents a measurable entity that the bedside clinician has available with which to estimate inflation energetic risk.

**Figure S1** Depiction of airway pressures over time and inflation energy during controlled ventilation with constant inspiratory flow. Areas quantify non-conserved (A) and two conserved energy blocks corresponding to driving pressure (B) and to PEEP (C). The filled area between the coarsely dashed line and the finely dashed slanting boundary of conserved pressure represents unrecovered (non-conserved) energy that corresponds to viscoelastic pressure losses with the possibility to apply directly damaging mechanical forces.

**Part B: Safety Ratios and Targets**

In the body of the paper, we gave expressions for ${HR}_{Drive}$ and ${HR}_{Elastic}$ from which derived expressions for the target driving pressure and target plateau pressure. Interrelationships among these variables are illustrated in Figure S2 If instead we use the safety ratio ($SR=1-HR$), it is possible to derive expressions for ${SR}_{Drive}$ and ${SR}_{Elastic}$, along with the corresponding $target DP$ and $target P_{s}$. In particular, one finds:

$${SR}_{Drive}= \frac{{{(P}_{t}-PEEP)}^{2}}{{(P_{s}-PEEP)}^{2}}=\frac{{{(P}_{t}-P_{s}+DP)}^{2}}{{DP}^{2}}$$

$${SR}_{Elastic}= \frac{{{P_{t}}^{2} - PEEP}^{2}}{{P_{s}}^{2}- {PEEP}^{2}}$$

from which we calculate

$$target DP=\frac{1-\sqrt{{SR}_{Drive}}}{1-{SR}_{Drive}}\left( P_{s}-P_{t} \right)$$

$$target P_{s}=\sqrt{\frac{{P_{t}}^{2} -{PEEP}^{2}}{{SR}_{Elastic}}+ {PEEP}^{2}}$$

**
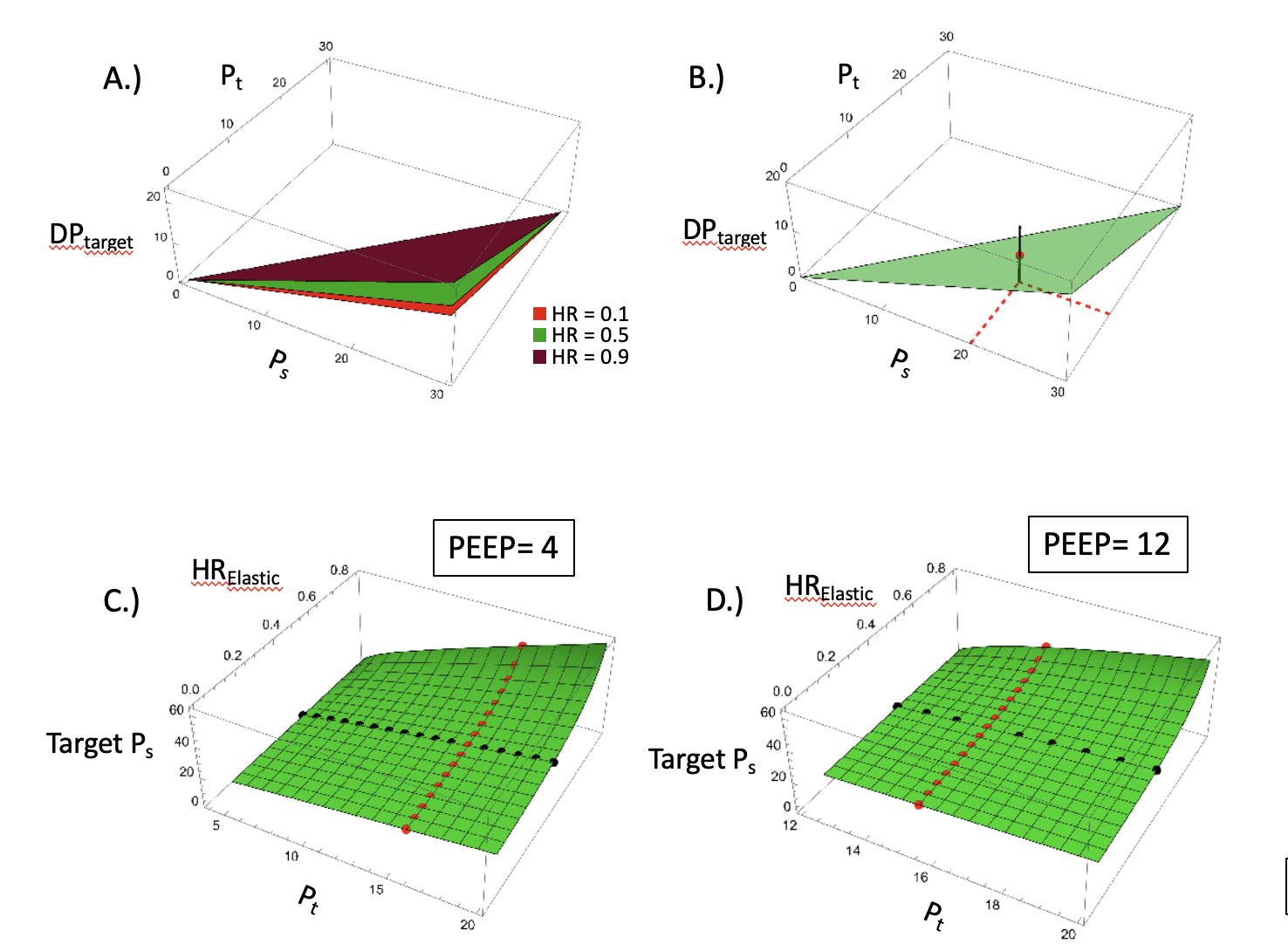
**

**____________________________________________________________________________**

**Figure S2:** Interrelationships among target values and hazard ratios. All pressures are expressed in cmH_2_O. Each value of ${HR}_{Drive}$ defines a unique three-dimensional (3D) plane (panel 3a), of which 3 are illustrated. Once a value for ${HR}_{Drive}$ is chosen, the value for the target driving pressure (*DP_target_*) can be computed in terms of the threshold pressure (*P_t_*) and plateau pressure (*P_s_*) (3b). In a similar fashion, each value of *PEEP* defines a 3D surface on which the target plateau pressure can be computed in terms of the threshold pressure and elastic hazard ratio ${(HR}_{Elastic}$ ) (3c,d).

**Part C: Estimating Regional Pt**

Utilizing the HR, SR, and targeting concepts and formulas elaborated in the text of the main paper, the range of *regional* vulnerabilities to tidal energy theoretically can be taken into account by adjusting the $P_{t}$ input by a fraction of the elastic pressure range that separates the *average* $P_{t}$ value from PEEP or $P_{s}$. Doing so would assign a locally relevant lower $P_{t}$ ($P_{t}$_lower_) or a higher $P_{t}$ ($P_{t}$_higher_), respectively (Figure 3 of main paper). A $P_{t}$_lower_ region (with stretch as the only criterion, for example, anatomically non-dependent) would be exposed to more than the average amount of total delivered hazardous energy during inflation [and thus is characterized by high vulnerability]; conversely, a $P_{t}$_higher_ region—e.g., anatomically dependent--would have less tidal energy delivered above its regional threshold [and thus is characterized by lower-than-average vulnerability]. Their corresponding HRs, SRs and targets would reflect those departures from the ‘average’ $P_{t}$.

Assuming that regional $P_{t}$ values vary from the average value over a ‘vulnerability’ range whose regional values deviate from the average $P_{t}$ by a factor of 𝛼 *or* β, the span of modified threshold pressures (high and low regional $P_{t}$ values) for that range would be:

$${P_{t}}_{lower}= P_{t} \left( 1-\alpha\right)+\alpha\left( PEEP \right), 0\leq\alpha\leq1$$

$${P_{t}}_{higher}= P_{t} \left( 1-\beta\right)+\beta(P_{s}), 0\leq\beta\leq1$$

When *regional* SRs are of interest, these modified $P_{t}$ values can then be used in the equations already developed in the text of the main paper to estimate the theoretical safety or hazard of the region-relevant proportion of the energy it receives during tidal inflation.
